# Supplementary material for: Antimicrobial prophylaxis does not improve post-surgical outcomes in SIV/SHIV-uninfected or SIV/SHIV-infected macaques (Macaca mulatta and Macaca fascicularis) based on a retrospective analysis
Source: PLoS One. 2022 Apr 20;17(4):e0266616. doi: 10.1371/journal.pone.0266616 (PMC9020680; doi:10.1371/journal.pone.0266616)
Supplement: Attachment — Submitted filename: Dr. Srishti Soni.docx [file pone.0266616.s001.docx]

**Manuscript number**: PONE-D-21-26281

**Title**: Antimicrobial prophylaxis does not improve post-surgical outcomes in SIV/SHIVuninfected or SIV/SHIV-infected macaques (Macaca mulatta and Macaca fascicularis)

**Comments to the authors**

Here authors want to examine whether antimicrobial prophylaxis influence the post surgical outcomes in SIV/SHIV-uninfected or SIV/SHIV-infected macaques. The study is important for surgery of patients or for research purpose. The findings will add some information in modern literature that deals with different surgical techniques. However, I have some concerns on the manuscripts.

Title: the title is to be modified. The authors should mention about the type of study: prospective or retrospective?

Abstract: The abstract should be brief (objective- methods-findings- final conclusion). Addition of excessive result data is not required

Introduction: page no 101-106: This section should be converted into generalized statements. It should not be restricted to ONPRC infectious disease facility as such type of surgeries are also practiced in other research centers of various countries.

**Materials and methods**

L 208-210: The results of this section should reflect on result section.

L237-241: authors have mentioned a list of complications. On what basis, authors identified the true complication? It is not clear.

Table 1 & 2: it is not clear how the authors calculated %, what about the raw data status?

Statistical analysis: Authors mostly describe about the software. Which statistical methods were used to examine the significant difference between the percentages?

A consort statement (study flow diagram) is required.

**Result**

L 257: Need a concise information about demographics of cases

The digit of *P* value should be uniform.

Throughout the result section, authors mentioned the no of observations repeatedly. It is not desirable. These should be depicted in table format. After that results should be presented as to- the- point.

**Discussion**:

L335-337: How antibiotics affect key immune parameters particularly cefazolin & cephalexin (L205-206) that were used in surgeries? Explain.

L 344-353: the repetitions of results in the discussion is not desirable.

L356-360/366-377: authors use some superfluous/redundant statements that does not required without suitable references.

Specific comments:

a) It may be true that antimicrobial prophylaxis had no significant effect on PLN biopsy post-operative complication rates in SIV/SHIV infected animals but suitable explanations are lacking here to support the statement “’ SIV/SHIV infected animals that received prophylactic antibiotics had higher complication rates post-laparoscopy”
b) In this study, true post-operative complication were identified as inappetence; moderate to severe inflammation, swelling or bruising; evidence of pain; incision discharge; surgical site abscessation; suture removal; incision ulceration; and/or incision dehiscence.
c) The Antimicrobial prophylaxis may influence the post operative inappetence by gut upset/ altering the gut microbiota as the authors mentioned in the manuscript. However, it not clear how antimicrobial prophylaxis influence the above mentioned other identified complications.

**Comments to the editor:**

**Final comment:** The manuscript may be considered after fully addressing comments by the authors and it needs minor revision
